# Supplementary material for: Development and validation of predictive workable weight equations for Brazilian older adult residents in long-term care institutions
Source: PLoS One. 2023 Jan 20;18(1):e0280541. doi: 10.1371/journal.pone.0280541 (PMC9858379; doi:10.1371/journal.pone.0280541)
Supplement: Attachment — Submitted filename: Response to reviewers.docx [file pone.0280541.s001.docx]

**Journal Requirements:**

**When submitting your revision, we need you to address these additional requirements.**

**1. Please ensure that your manuscript meets PLOS ONE's style requirements, including those for file naming. The PLOS ONE style templates can be found at**

[**https://journals.plos.org/plosone/s/file?id=wjVg/PLOSOne_formatting_sample_main_body.pdf**](https://journals.plos.org/plosone/s/file?id=wjVg/PLOSOne_formatting_sample_main_body.pdf) **and** [**https://journals.plos.org/plosone/s/file?id=ba62/PLOSOne_formatting_sample_title_authors_affiliations.pdf**](https://journals.plos.org/plosone/s/file?id=ba62/PLOSOne_formatting_sample_title_authors_affiliations.pdf)**.**

A: The manuscript was adjusted according to the Plos One styles requirements.

**2. Please provide additional details regarding participant consent. In the ethics statement in the Methods and online submission information, please ensure that you have specified (1) whether consent was informed and (2) what type you obtained (for instance, written or verbal, and if verbal, how it was documented and witnessed). If your study included minors, state whether you obtained consent from parents or guardians. If the need for consent was waived by the ethics committee, please include this information.**

**If you are reporting a retrospective study of medical records or archived samples, please ensure that you have discussed whether all data were fully anonymized before you accessed them and/or whether the IRB or ethics committee waived the requirement for informed consent. If patients provided informed written consent to have data from their medical records used in research, please include this information.**

A: Details of participant consent were given in the Methods and online submission information.

**3. In your Data Availability statement, you have not specified where the minimal data set underlying the results described in your manuscript can be found. PLOS defines a study's minimal data set as the underlying data used to reach the conclusions drawn in the manuscript and any additional data required to replicate the reported study findings in their entirety. All PLOS journals require that the minimal data set be made fully available. For more information about our data policy, please see** [**http://journals.plos.org/plosone/s/data-availability**](http://journals.plos.org/plosone/s/data-availability)**.**

**"Upon re-submitting your revised manuscript, please upload your study’s minimal underlying data set as either Supporting Information files or to a stable, public repository and include the relevant URLs, DOIs, or accession numbers within your revised cover letter. For a list of acceptable repositories, please see http://journals.plos.org/plosone/s/data-availability#loc-recommended-repositories. Any potentially identifying patient information must be fully anonymized.**

**Important: If there are ethical or legal restrictions to sharing your data publicly, please explain these restrictions in detail. Please see our guidelines for more information on what we consider unacceptable restrictions to publicly sharing data: http://journals.plos.org/plosone/s/data-availability#loc-unacceptable-data-access-restrictions. Note that it is not acceptable for the authors to be the sole named individuals responsible for ensuring data access.**

**We will update your Data Availability statement to reflect the information you provide in your cover letter.**

A: The data is available and can be found at the link provided in the online submission.

**4. Please ensure that you include a title page within your main document. You should list all authors and all affiliations as per our author instructions and clearly indicate the corresponding author.**

A: Title page according to Plos One style was attached during online resubmission.

**Reviewers' comments:

Reviewer's Responses to Questions**

**Comments to the Author

1. Is the manuscript technically sound, and do the data support the conclusions?

The manuscript must describe a technically sound piece of scientific research with data that supports the conclusions. Experiments must have been conducted rigorously, with appropriate controls, replication, and sample sizes. The conclusions must be drawn appropriately based on the data presented.**

**Reviewer #1: Partly**

**Reviewer #2: Yes**

**2. Has the statistical analysis been performed appropriately and rigorously?**

**Reviewer #1: Yes**

**Reviewer #2: Yes**

**3. Have the authors made all data underlying the findings in their manuscript fully available?

The** [**PLOS Data policy**](http://www.plosone.org/static/policies.action#sharing) **requires authors to make all data underlying the findings described in their manuscript fully available without restriction, with rare exception (please refer to the Data Availability Statement in the manuscript PDF file). The data should be provided as part of the manuscript or its supporting information, or deposited to a public repository. For example, in addition to summary statistics, the data points behind means, medians and variance measures should be available. If there are restrictions on publicly sharing data—e.g. participant privacy or use of data from a third party—those must be specified.**

**Reviewer #1: No**

**Reviewer #2: Yes**

**4. Is the manuscript presented in an intelligible fashion and written in standard English?

PLOS ONE does not copyedit accepted manuscripts, so the language in submitted articles must be clear, correct, and unambiguous. Any typographical or grammatical errors should be corrected at revision, so please note any specific errors here.**

**Reviewer #1: No**

**Reviewer #2: Yes**

**5. Review Comments to the Author

Please use the space provided to explain your answers to the questions above. You may also include additional comments for the author, including concerns about dual publication, research ethics, or publication ethics. (Please upload your review as an attachment if it exceeds 20,000 characters)**

**Reviewer #1: The study is really necessary study since the authors address a very important aspect of health of older people. I have few concerns with this study.
1. Better to exclude the 55 individuals who participated for the study at 1st time from the 2nd stage and re analyze the data.**

A: Done. Results did not change.

**2. There should be a coherence in the writing specially the methods, data analysis, results and discussion.**

A: Sections were adjusted.

**3. Methods – study design, period of data collection, stage I and 2, way of taking anthropometry information, data analysis and ethical clearance.**

A: Sections were adjusted.

**4. Data analysis – how did you present the descriptive data, removing outliers, comparing the characteristics of two group (development and validation groups; there should not be difference), stratification, correlation analysis (between weight and anthropometry), regression analysis to choose the model/equations, Bland-Altman plots, comparing with previous study equations etc.**

A: Sections were adjusted.

**5. Results should be arranged in the same sequence of results analysis.**

A: Done. Sections were adjusted.

**6. Discussion – summary of findings, reasons for observations, similarities and discrepancies with the existing literature, reasons for similarities and discrepancies, applications of these findings, strengths and limitation, recommendations.**

A: Section was adjusted.

**7. Need language improvement.**

A: Language improvement was done.

**Reviewer #2: This is a relevant topic since in older adults’ weight is the anthropometric measure that best relates to malnutrition, especially if there are changes from baseline weight. Furthermore, weight must be known in order to use tools such as body mass index (BMI) and the Mini Nutritional Assessment test (MNA) for nutritional screening. However, in hospitalized or institutionalized older adults, it is not always possible to determine weight on a conventional scale. Therefore, it is important to find equations that could serve to perform screening for malnutrition.
The goal of the work is clearly described.
The work presented is ethically sound. Authors have submitted the research protocol for approval by an University ethics committee.

Older individuals become more heterogeneous with age, a specific descriptor such as elderly is inaccurate and misleading thus authors should consider the replacement of “elderly” by “older adults” in all the manuscript .**

A: Done. It was replaced “elderly” by “older adults” in all the manuscript.

**Title: The title should mention “older adults”**

A: Done. Title has the term “older adults”.

**Introduction: In the 3th paragraph last sentence “As such,……” should be rewritten**

A: Done.

**Methods: Inclusion and exclusion criteria of the patients should be presented**

A: Done.

**Results
Table2- Authors should correct the legend in order to consider the arm circumference (AC)**

A: Done. Thanks for attention.

**In the last paragraph of the discussion the abbreviations ICC and CI should be described: (Intraclass Correlation Coefficient (ICC)………..)**

A: Done.  **Discussion: Authors might hypothesise about the concordance of their models with those of Chumlea et al. (1988) and Rabito et al. (2008) .**

A: Done.

**Considering the above comments, I recommend that manuscript could be accepted with minor revisions**

**6. PLOS authors have the option to publish the peer review history of their article ([what does this mean?](https://journals.plos.org/plosone/s/editorial-and-peer-review-process#loc-peer-review-history)). If published, this will include your full peer review and any attached files.


If you choose “no”, your identity will remain anonymous but your review may still be made public.


Do you want your identity to be public for this peer review? For information about this choice, including consent withdrawal, please see our** [**Privacy Policy**](https://www.plos.org/privacy-policy)**.**

**Reviewer #1: No**

**Reviewer #2: No**

**[NOTE: If reviewer comments were submitted as an attachment file, they will be attached to this email and accessible via the submission site. Please log into your account, locate the manuscript record, and check for the action link "View Attachments". If this link does not appear, there are no attachment files.]

While revising your submission, please upload your figure files to the Preflight Analysis and Conversion Engine (PACE) digital diagnostic tool,** [**https://pacev2.apexcovantage.com/**](https://pacev2.apexcovantage.com/)**. PACE helps ensure that figures meet PLOS requirements. To use PACE, you must first register as a user. Registration is free. Then, login and navigate to the UPLOAD tab, where you will find detailed instructions on how to use the tool. If you encounter any issues or have any questions when using PACE, please email PLOS at** [**figures@plos.org**](mailto:figures@plos.org)**. Please note that Supporting Information files do not need this step.**
