# Supplementary material for: Development and validation of predictive workable weight equations for Brazilian older adult residents in long-term care institutions
Source: PLoS One. 2023 Jan 20;18(1):e0280541. doi: 10.1371/journal.pone.0280541 (PMC9858379; doi:10.1371/journal.pone.0280541)
Supplement: Attachment — Submitted filename: Response to reviewers.docx [file pone.0280541.s002.docx]

**6. Review Comments to the Author

Please use the space provided to explain your answers to the questions above. You may also include additional comments for the author, including concerns about dual publication, research ethics, or publication ethics. (Please upload your review as an attachment if it exceeds 20,000 characters)**

**Reviewer #2: Authors have respond to all the questions adressed. Moreover they have improved the language in order to accomplish the concerns of the reviewers. Considering the above manuscript should be accepted.**

**Reviewer #3: The manuscript presents an interesting topic, with relevance for clinical practice.
However, there are still issues that need to be clarified:

- Methods: please provide the range of values for the technical error of measurement.**

**Done.**

**- Results: please explain the sentence: "The characterization of the study population showed that the anthropometric measurements taken differed in the groups studied".**

**The sentence was changed for “The characterization of the study population showed that the anthropometric measurements were different for each studied group”.**

**- The conclusion should contain an objective response to the aim of the study.**

**Done. It was added to conclusion “A set of five equations for estimating weight were developed and validated”.**

**- A general review of the English language is suggested throughout the text.**

**Done. Language was improved by translate review.**
