# Supplementary material for: Development and validation of predictive workable weight equations for Brazilian older adult residents in long-term care institutions
Source: PLoS One. 2023 Jan 20;18(1):e0280541. doi: 10.1371/journal.pone.0280541 (PMC9858379; doi:10.1371/journal.pone.0280541)
Supplement: Attachment — Submitted filename: Response to reviewers(3).docx [file pone.0280541.s003.docx]

**6. Review Comments to the Author

We've checked your submission and before we can proceed, we need you to address the following issues:**

**1. Please amend the title either on the online submission form or in your so that they are identical.**

**Title changed on the online submission form for “Development and validation of predictive workable weight equations for Brazilian older adult residents in Long-Term Care Institutions” according to manuscript**.
